# Corneal Sublayers Thickness Estimation Obtained by High-Resolution FD-OCT

**DOI:** 10.1155/2013/989624

**Published:** 2013-06-02

**Authors:** Diego Alberto, Roberto Garello

**Affiliations:** ^1^Department of Electronics and Telecommunications, Politecnico di Torino, Corso Duca Degli Abruzzi 24, 10129 Turin, Italy; ^2^TIMA Laboratory (Grenoble INP, UJF, CNRS), 46 avenue Félix Viallet, 38031 Grenoble, France

## Abstract

This paper presents a novel processing technique which can be applied to corneal in vivo images obtained with optical coherence tomograms across the central meridian of the cornea. The method allows to estimate the thickness of the corneal sublayers (Epithelium, Bowman's layer, Stroma, Endothelium, and whole corneal thickness) at any location, including the center and the midperiphery, on both nasal and temporal sides. The analysis is carried out on both the pixel and subpixel scales to reduce the uncertainty in thickness estimations. This technique allows quick and noninvasive assessment of patients. As an example of application and validation, we present the results obtained from the analysis of 52 healthy subjects, each with 3 scans per eye, for a total of more than 300 images. Particular attention has been paid to the statistical interpretation of the obtained results to find a representative assessment of each sublayer's thickness.

## 1. Introduction 

Optical coherence tomography (OCT) based on low coherence interferometry is a well-established imaging technique thanks to its prominent axial resolution. Since 2006, commercially available OCT systems perform visualization of tissue microstructure in the so-called Fourier Domain (FD-OCT). Differently to Time Domain OCT (TD-OCT), the whole depth structure is obtained synchronously providing higher resolution imaging with faster acquisition times. FD-OCT can be used to provide in vivo cross-sectional imaging of the eye in a noninvasive and noncontact way [1]. To date, this technique has been mostly applied to capture retinal structure and optic nerve, displaying and localizing discrete morphological changes in detail [2, 3].

In this paper we use an FD-OCT to study the anterior segment of the eye since this acquisition system can produce cross-sectional images of the cornea, which can be properly processed to analyze corneal sublayers: Epithelium, Bowman's layer, Stroma, Descemet's membrane, and Endothelium [4–7]. The precise measurement of these sublayers thickness is very important in ophthalmics and optometrics, for therapeutic treatments, refractive surgery, and contact lens applications. 

Many works have presented the thickness estimation of corneal sublayers techniques different from OCT. All these approaches have drawbacks and/or introduce some restrictions. Confocal microscopy [8] is an invasive technique that can cause lesions of corneal tissues, while electron microscopy [8, 9] deals only with histopathologic samples, and ultrasonic pachymetry [10] requires the instillation of a topical anaesthetic and well trained operators. 

On the contrary, OCT has the advantage of allowing quick, noninvasive and completely safe assessment of patients [2, 3]. Unfortunately, traditional image processing techniques, such as Sobel or Canny algorithms [11], failed in boundary localization of OCT cross-sections because, in general, these images present low Signal-to-Noise Ratio (SNR) and low contrast between boundary and internal corneal regions [5–7].

In this work, a novel technique based on automated edge detection method is presented. This procedure, based on SNR enhancement and corneal sublayers segmentation, allows to accurately extract the sublayers thickness information from FD-OCT images.

## 2. Material and Methods

### 2.1. FD-OCT Corneal In vivo Images and Sublayer Thickness Estimation Problem

**Figure 1:**
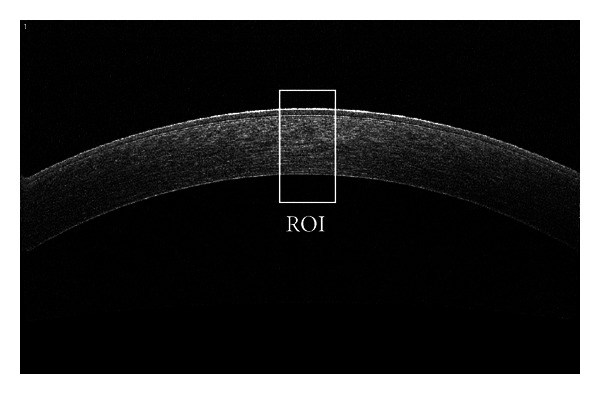
OCT grayscale corneal tomogram and the region of interest (ROI) on the corneal apex. (in this case: horizontal dimension: 6 mm, 1016 pixels; vertical dimension: 4 mm, 640 pixels.)

A FD-OCT corneal in vivo image appears as in Figure 1. Different reflectivity profile boundaries identify different corneal sublayers, as reported in histological examinations [5]. 

Since each image is available in grayscale format and is uploaded as a matrix of pixels [5, 6], the pixel intensity can be treated as the third coordinate. This reflectivity profile information can be considered as the amplitude of the signals to be constructed and analyzed, see Figure 1.

**Figure 2:**
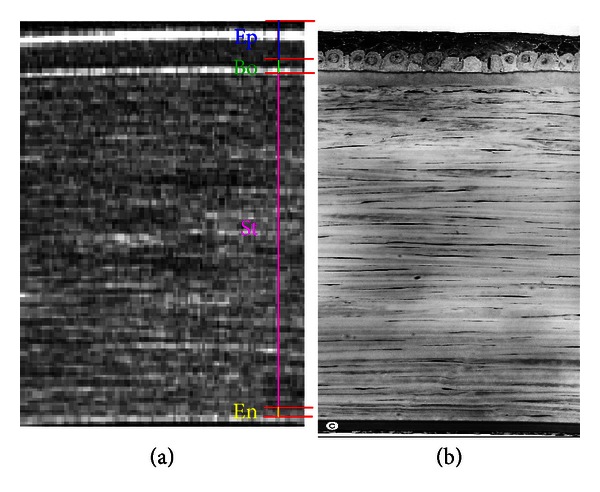
(a) Details of the ROI of Figure 1 with sublayers subdivision: Epithelium (Ep), Bowman (Bo), Stroma (St), and Endothelium (En). The Descemet's membrane is not reported. (b) Histological sample of a human cornea (the two pictures do not belong to the same patient).

In more detail, the region of interest (ROI) highlighted in the Figure 1 is shown in Figure 2(a). As a reference, in Figure 2(b), a human corneal histological sample is presented and graphically compared with Figure 2(a). (The two pictures do not come from the same subject.)

Human corneas, like those of other primates, are composed of five sublayers: *Epithelium* is a layer of cells that cover the surface of the cornea; *Bowman's layer* protects the Stroma from injury; *Stroma *is the thickest layer (90% of the corneal thickness), transparent and made of collagen fibrils; *Descemet's membrane* is the thinnest layer, only one cell thick (too thin to be detected [5] and also in our study it will not be estimated); *Endothelium* is a low cuboidal monolayer of mitochondria-rich cells. 

The knowledge of their thicknesses is of significant importance for ophthalmics and optometrics examinations and treatments.

To validate the proposed analysis protocol, a sample of 52 healthy patients has been considered: 25 females and 27 males, mean ± standard deviation age: 34 ± 11 years, range: 25 to 74 years. All the subjects did not present any ocular disease nor any history of ocular surgery and have been analyzed with the FD-OCT system described in Section 2.2. This group of patients can be defined as *normal* patients. In this paper, the analyses of the only right eyes are reported since no significant difference occurred in the comparison between right and left eyes, nor between male and female subgroups (for the statistical validation see Section 4).

The considered problem consists of estimating the thickness of each sublayer starting from images like that depicted in Figure 1. Unfortunately, these images are characterized by low SNR values which prevents the application of classical techniques. 

Even if the algorithm is presented in general and can be applied to any starting FD-OCT image, we provide here all the details of the experimental set-up to allow reproducibility of the results and better understand the analysis scenario. 

### 2.2. Experimental Setup and Acquisition Procedure

A FD-OCT RTVue-100 Optovue device [12] was used. The reflectivity profile (A-scan) information was acquired by a CCD camera simultaneously. Due to the fast CCD camera line transfer rate and fast Fourier transform algorithm, this FD-OCT could perform 26000 A-scan/second. Each tomogram was the average of 16 images. The Super Luminescent Diode (SLD) this device provided worked at a wavelength of 840 ± 10 nm. It was connected to a telecentric light delivery system and mounted on a standard slit-lamp. This wavelength value was adopted since it allowed higher SNR than older OCT devices [13]. Furthermore, it was already chosen to analyze retinal imaging [14] and to obtain higher axial resolutions with the same bandwidth. Corneal imaging was performed with auxiliary lens (CAM-l), helpful for corneal structures magnification. 

The working distance between patients and the OCT device was 22 mm. Subjects were asked to put their chin on the slit-lamp and to watch the target in the central point of the OCT probe. The exposure power at pupil was 750 *μ*W. This low value guaranteed no damage to analyzed eyes being below the maximum permissible exposure dictated by the American National Standards Institute (ANSI) at this wavelength [15]. The axial calibration of the OCT was performed using a set of polymethylmethacrylate (PMMA) lenses of known thickness (546 ± 1 *μ*m) and constant index of refraction (1.4838 at 840 nm) [16, 17]. The PMMA lenses were measured using a Mitutoyo micrometer [18, 19]. The FD-OCT declared resolutions were: 5.0 *μ*m in depth (axial direction), and 15 *μ*m for the transverse direction [12]. The investigated corneal area was 6 mm × 4 mm, corresponding to a matrix of 1016 × 640 pixels. Simply performing the division between these correlated values, the axial resolution is equal to 6.25 *μ*m for and the transverse one to 5.91 *μ*m. To find a more reliable pixel-*μ*m conversion factor, a calibration procedure was applied. By examining 10 OCT images of a set of PMMA contact lenses with known thickness and index of refraction, the conversion factor pixel-*μ*m has been found: 1 pixel = 4.13 *μ*m, 1 subpixel = 0.52 *μ*m [16–18] for axial resolution (for the pixel-subpixel chosen ratio see Section 4). These were the mean values of the conversion factors obtained from the analysis carried on the complete set of lenses. As a simplification, it was decided to consider neither the deterministic nor the statistical errors performed both in PMMA lens thickness estimation and in their OCT acquisitions to avoid their propagations. Unfortunately, with OCT acquisitions of PMMA lenses it was not possible to estimate the transverse resolution, therefore, 5.91 *μ*m/pixel was assumed. The difference between the chosen axial and transverse resolutions is close to the one presented in [6]. 

The OCT was connected to a computer to visualize and store corneal images. In a second analysis, the acquired tomograms were processed to extract the features of interest. The average time duration per patient of the medical analysis was 10 minutes, whereas the digital processing required only few seconds. 

## 3. The Algorithm for Estimating the Sublayer Thickness: Estimation Problem

In an OCT corneal tomogram the cornea and its internal sublayers are represented by different grayscale regions since each corneal tissue presents a different reflectivity, see Figures 2(a) and 2(b). In particular, the boundary between two consecutive sublayers presents a constant reflectivity profile [5, 6]. Enhancing the SNR of each analyzed region, our algorithm quickly detects these boundaries (edges) and estimates the sublayer thicknesses evaluating the distance between two consecutive couple of edges. 

**Figure 3:**
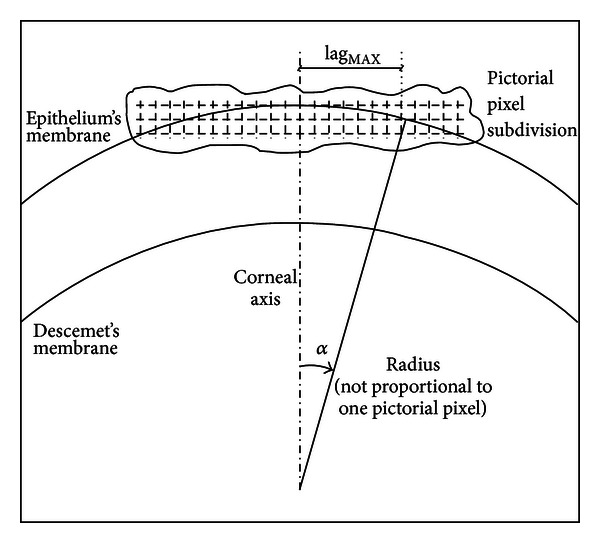
Pictorial representation of the procedure used to estimate the maximum number of columns on which the pixel intensity analysis is performed. In this sketch, the drawn Radius is not proportional to the pictorial pixel dimension.

As a first step, we need to identify on the tomogram the dimension and direction of the ROI to be analyzed, as in Figure 2(a). Due to the natural shape (curvature) of the cornea, particular attention must be paid to the chosen region. Taking for instance into account the central region of the cornea and working symmetrically on the apex of every meridian, it is mandatory not to consider pixels from different sublayers on the same row, see Figure 3. This kind of problem could arise if we consider too wide regions. The procedure for determining the ROI maximum dimension, denoted as the 2lag_MAX_ value, is depicted in Figure 3. As a result, this region can be assumed straight, or affected by negligible corneal curvature, and every sublayer represented on the same pixel row. 

In our case study, the ROI maximum dimension chosen according to this rule has been found, on average, equal to 2lag_MAX_ = 90 pixels (~532 *μ*m, for the transverse pixel-*μ*m conversion see Section 2.2).

**Figure 4:**
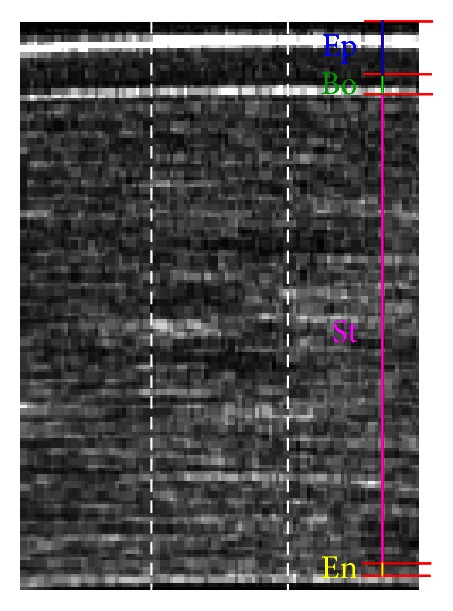
The four sublayers to be estimated with the ROI subdivision into three adjacent slides.

As a second step, the ROI area is divided into three slices one next to the other, see Figure 4. Each slice is composed of the same number of pixel columns (25–30 in our case study), depending on the 2lag_MAX_ value chosen in the previous step. These three slices, being adjacent, are considered not to be affected by significant differences of thickness. Note that this subdivision into three slices is essential also for the statistical validation (Section 4) of the presented approach.

With reference to the histological model (Figure 2(b)), corneal sublayers can be identified from the different reflectivity profile of the anatomical boundaries. Suppose that a slice is composed of one (central) pixel column. Different reflectivity is mapped by a proportional pixel grayscale intensity, valued between 0 and 255 [5, 6]. If the intensity depth profile is plotted as a function of pixel rows, the search of minima and maxima values leads to the localization of the beginning of corneal sublayers. In order to reduce noise (flicker, speckle, etc.), the pixel intensity reflectivity profile is linearly averaged on the number of columns composing the slice (25–30 in our case study). We will refer to this procedure as the *averaging technique*. This procedure is justified by the evidence that, inside each ROI slice, pixel rows of the same region show a Gaussian reflectivity profile distribution (Pearson's chi-square test [20], *P* < 0.05). 

The same sublayer boundary shows nearly the same reflectivity index, and it is represented by a continuous line in an OCT tomogram (see Figures 2(a) and 2(b)). Conversely, regions inside two consecutive boundaries present nonconstant reflectivity values (due to the anatomy of the analyzed tissues [5]). In the current analysis, this behavior can be considered as additive noise that makes it harder to find peak values that delimit sublayer regions. The average of pixel intensity values performed on near columns can preserve the boundary pixel values and reduce the uncertainty introduced by pixels coming from inner regions. The robustness of this procedure can be estimated by comparing the SNR evaluated on a single column with the one calculated on averaged columns (see Section 4). 

**Figure 5:**
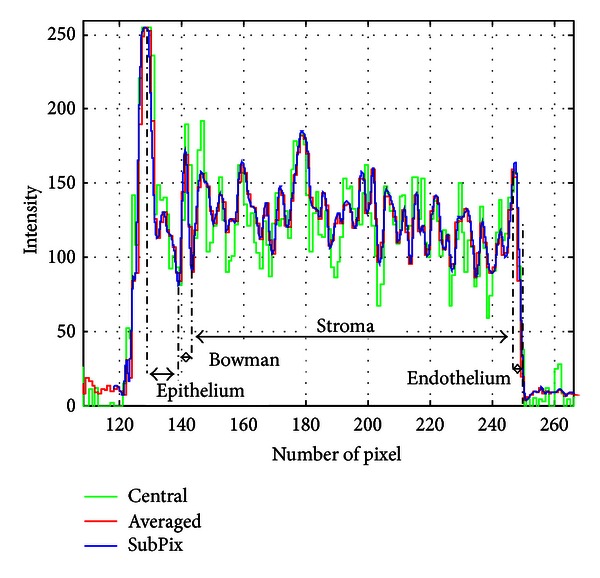
Pixel intensity profile on a 25 pixels wide slice, centred on the corneal apex. In green the intensity of the only axial column is represented, while in red and blue, the averaged intensities in pixel and subpixel elaborations, respectively.

**Figure 6:**
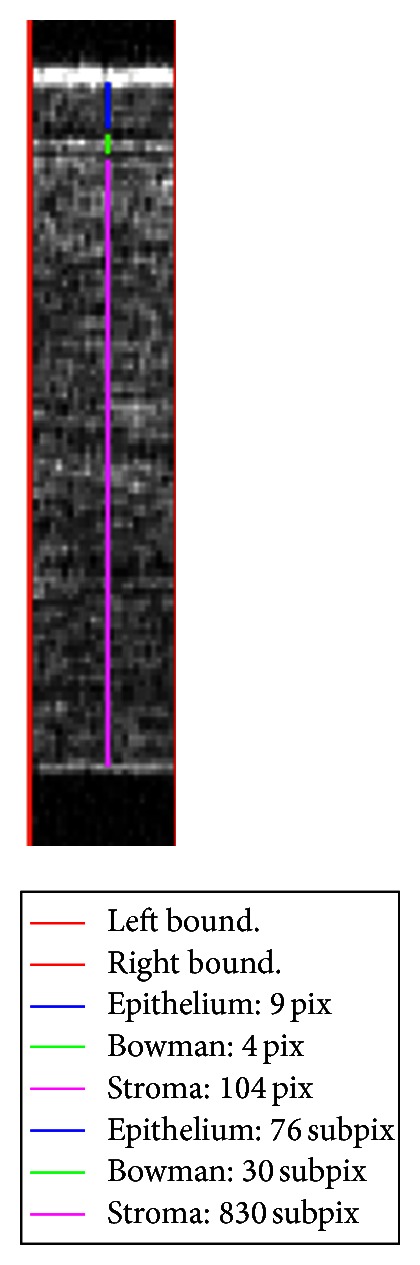
Detail of the central ROI slice. In the box, thickness estimates are reported both in pixel and subpixel scale.

Figures 5 and 6 show the results of this procedure; for the pixel-subpixel chosen ratio see the following section. Epithelium, Bowman's layer, Stroma, and Endothelium sublayers can be identified. The global maximum corresponds to the anterior surface of Epithelium; the global minimum is the end of the Epithelium. The Bowman's layer starts immediately after the end of the Epithelium and it ends at the following (second) global minimum. The Stroma starts after the second minimum and ends at the last maximum on the right side of the pixel intensity profile. Endothelium starts after the last maximum and its end is assumed (approximation) where the signal goes under two RMS of pixel intensity values coming from the right region outside cornea (noise). Descemet's membrane is too thin to be detected being, if present, nearly or less than one pixel width [21].

A customized MATLAB [22] program has been developed to automatically process all the images and accurately segment the inner corneal sublayers. All analyses have been carried out using a MATLAB platform. 

### 3.1. Subpixel Procedure

The approach described in the previous section has been carried out on three slices (see Figure 2(b)) of the same ROI to evaluate the uncertainties on the sublayer thickness estimations by means of weighted averages. It is worth noting that in using the automated procedure in pixel scale, the original image is not altered. As a further step, a subpixeling technique has been applied to reduce the uncertainty in thickness estimations when measurements were expressed in pixel scale. It was obtained with a bi-cubic interpolation [22, 23] on both image directions and was not a super-resolution technique. It simply helped in the statistical analysis to distinguish cases that presented the same thickness value estimated in pixel scale for two, or all three, slices of the same ROI. Interpolating does not increase axial/transverse resolution but can increase only digital resolution of the image. The chosen linear ratio between pixel and subpixel has been set one to eight. For ease of elaboration it had to be a power of two [22], and the value eight was the first that highly reduced the aforementioned cases that presented the same estimated thickness. As a result, the subpixel intensity profile is smoothed if compared with the pixel averaged sample, as shown in Figure 5. 

### 3.2. Application to Different Regions

**Figure 7:**
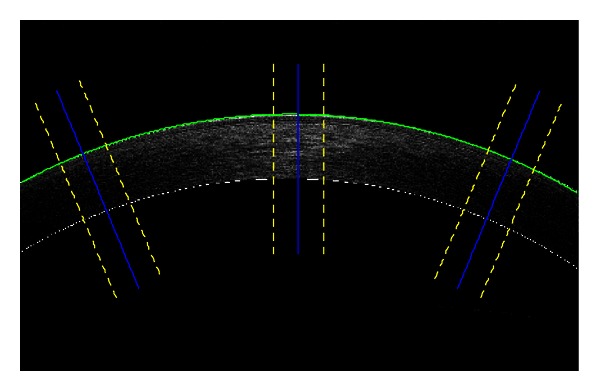
Corneal image of one of the analysed patients with the interpolated air-Epithelium boundary (green curve). Central and midperipheral (nasal and temporal) axes are represented (blue solid lines) together with the delimitations of the three ROIs (yellow dashed lines). The investigated midperipheral angle is 23 degrees in both sides.

Interpolating the air-Epithelium boundary and working orthogonally to this sublayer, the procedure described in Section 3 can be also utilized to study midperipheral corneal regions, both nasal and temporal sides, see Figure 7. For the same reason discussed in the previous section, in order not to consider pixels coming from different sublayer on the same analyzed line (orthogonal to the investigated axes, blue solid segment in Figure 7), the width of the analyzed marginal ROI must be properly chosen (about 85–90 pixels in our case study), in accordance with the estimated curvature of the considered tomogram. With this approach, a more detailed investigation of the corneal sublayers thickness behavior can be obtained. 

In the literature, polynomial approximations of corneal sublayers' two dimensional profiles are widely assumed [6, 24]. In this work, however, a circumference has been preferred since this curve provides not only a good agreement with analyzed data (Pearson's chi-square goodness-of-fit test [20], *P* < 0.05), but also a reference (its center) to measure the midperipheral nasal and temporal angles at which the aforementioned method can be applied, see Figure 7.

In principle, every patient's tomogram could be analyzed on every marginal region of the corneal image. In practice, the SNR of the considered subregion limits the application of our procedure. For example, in Figure 7 the tomogram has been investigated at 23 degrees on both nasal and temporal sides. This chosen angle represents the highest value at which the averaging technique returns signals with SNR high enough to be efficiently analyzed. The obtained results have been reported and compared in the following section. 

## 4. Study Results

To validate our technique, the procedure described in the previous section has been applied to the sample of 52 healthy patients reported in Section 2.1.

A statistical analysis of the proposed procedure is mandatory to validate our methodology and to build a reference of sublayer thickness values for normal patients. 

For every subject, three images of each eye have been recorded on a corneal area of 6 mm × 4 mm (1016 × 640 pixels), for a total number of 312 images, see one of them in Figure 1.

Even though FD-OCT devices present very good repeatability and accuracy [25], to give an additional validation to the reported measurements, the estimated sublayers' thickness (in pixel scale) were compared, fixed the region (nasal, central or temporal), on the first and on the third acquisition of the same set of patients for the right eye. No significant differences have been identified (paired samples *t*-test [20], *P* < 0.05).

The complete set of images has been processed with the customized algorithm introduced in Section 3. On the apex and on the midperipheral nasal and temporal sides of the horizontal meridian, the resulting thicknesses of all sublayers and of the total cornea are reported in Table 1. It is worth recalling that the small uncertainties are due to the weighted average procedure.

The sublayer estimations of central corneal thicknesses are all in accordance with the results presented in [5] except for the Epithelium, where the difference of nearly 10 *μ*m is clearly significant if compared with the standard deviations (unpaired samples *t*-test [20], *P* < 0.05). A possible explanation of this discrepancy can be found in the way the Epithelium starting point was defined in Section 3. It was assumed to be the first pixel after the global reflectivity maximum. This highest value is due to the presence of tears and can be a plateau two or three pixels wide (corresponding to 10–12 *μ*m).

Midperipheral corneal thicknesses cannot be strictly compared with the results proposed in [5] since they refer to regions that differ by 3 degrees (angle separation). However, they remain statistically compatible (unpaired samples *t*-test [20], *P* < 0.05) and the difference between the Bowman's layer estimates is due to the same reason explained for the central thickness analysis. Total corneal increasing behavior is evident, as reported also in [6]. Furthermore, the contribution to the TCT increment is due to Epithelium and Stroma, while Bowman's layer remains nearly constant, in accordance with [5].

As introduced in Section 2.1, this paper reports the results of the only right eye (Table 1), since no significant difference occurred in the comparison between right and left eyes, nor between male and female subgroups (resp. paired and unpaired samples *t*-test [20], *P* < 0.05). These statistical results are in accordance with the previously reported study [5].

A useful feature in the processing of an OCT corneal image is represented by the SNR improvement obtained using the previously discussed averaging technique. The noise component was assumed to be studied in the only Stromal region since it is the thickest among all corneal sublayers with an average estimated thickness of the order of 100 pixels on the corneal apex.

By carrying out this analysis on the central ROI (Figure 7) in pixel scale and evaluating the SNR on the subset of images coming from the third acquisition of the right eye for all 52 patients, in every image the SNR distributions of the central pixel column and of the averaged columns composing the central slice were compared. In both cases the values were Gaussian distributed (Pearson's chi-square test [20], *P* < 0.05). The central column presented a SNR mean value equal to 6.7 dB and a RMS of 0.8 dB, while the averaged signal showed a mean value of 13.9 dB and a RMS of 1.7 dB. Taking into account the mean values of these two distributions, the improvement obtained with the averaging technique was 7.2 dB. This rise allowed an accurate and robust sublayer boundaries detection and consequent thickness estimations. 

By applying the same SNR estimate procedure to midperipheral corneal regions (Figure 7) of the same subset of images, SNR values remained Gaussian distributed (Pearson's chi-square test [20], *P* < 0.05). However, central midperipheral axes presented a SNR mean value reduced to 3.7 dB and a RMS of 1.2 dB, while the signals averaged on peripheral ROIs showed a mean value of 9.4 dB and a RMS of 2.0 dB. Considering also in this case the only mean values, the improvement obtained with the averaging technique was 5.7 dB. This rise confirmed the validity of the averaging procedure but, the absolute SNR value of processed signals was the lowest able to allow an accurate and robust sublayer boundaries detection. This is the reason why more peripheral corneal regions cannot be processed with this approach on the considered tomograms. Note that these SNR evaluations on central and peripheral ROIs come from images in pixel resolution since no significant difference has been found carrying out this analysis in subpixel scale (paired samples *t*-test [20], *P* < 0.05).

## 5. Discussion 

The number of patients considered in this work was more than double the ones presented in [5, 6, 21, 24]. All subjects gave the informed consent to the collection and use of recorded data and were treated according to the tenets of the Declaration of Helsinki.

Whereas the algorithm itself and the estimated results can be considered validated, the case study and its medical reliability clearly present some minor limitations. Firstly, the database was composed of 52 patients only and may be the reason why no significant age or genders differences have been identified (statistical problem). Secondly, the device returned few cases of *too dark* OCT corneal images that presented a SNR low enough to make any processing impossible, especially in midperipheral sides. In such cases new images from the same patients were acquired. Thirdly, Descemet's membrane was not distinguishable, as in the work reported in [5]. This sublayer, if present, could vary the Stroma thickness estimations of 10–15 *μ*m for people without any ocular disease [21]. Fourthly, a precise estimate of the OCT transverse resolution was not obtained, however, it can be determined using, for example, special USAF targets [26]. To quantify the influence of this uncertainty at the considered midperipheral angle and with the procedure described in Section 3.2, a variation of ±0.5 *μ*m on the transverse resolution leads to a variation of ±2.2% in the midperipheral results reported in Table 1. Notwithstanding that, they still remain in accordance with [5].

Finally, because of this technology, the ROI regions were assumed straight or affected by negligible curvature. 

## 6. Conclusions

A robust technique for estimating corneal sublayer thickness starting from low-SNR FD-OCT images has been presented and validated by statistical analysis. The introduced procedure allowed a significant SNR enhancement and an analysis on a wide region of the considered OCT tomograms.

The method has been utilized for the study of sublayer thickness estimations on a sample of 52 healthy patients without any optical disease on both central and peripheral regions of the horizontal meridian of the cornea by using more than 300 FD-OCT corneal images. 

From this analysis, the average values for all sublayers in three different corneal regions have been provided and, fixed the corneal area, no significant difference between right and left eyes or between male and female subgroups occurred. In addition, an averaging technique has been introduced to construct reference signals to be used for thickness estimations. The improvement in SNR has been introduced and discussed. The method is very useful to provide a fast, simple but robust and noninvasive estimation of the sublayer thickness. Its advantages and limitations have been discussed in the paper.

As future work, a USAF target can be imaged to estimate OCT transverse resolution. Furthermore, the processing of corneal marginal regions can be applied to study medical cases in order to quantify the effective change in corneal sublayers produced by ophthalmological treatments. Finally, further development is in progress for studying a special class of customized contact lens thickness estimations.

## Figures and Tables

**Table 1:** Mid-peripheral nasal, central, and mid-peripheral temporal corneal sublayers thickness estimation obtained averaging the respective evaluations performed on 156 images (right eye). The Total corneal thickness (TCT) value is calculated as the sum of all sublayers.

Sublayer	Midperiph. nasal (*μ*m)	Central (*μ*m)	Midperiph. temp. (*μ*m)
Epithelium	45.3 ± 0.5	42.8 ± 0.5	45.1 ± 0.5
Bowman	17.2 ± 0.5	17.0 ± 0.5	16.9 ± 0.5
Stroma	477.0 ± 0.5	453.0 ± 0.5	473.0 ± 0.5
Endothelium	10.1 ± 0.5	9.8 ± 0.5	9.9 ± 0.5
TCT	550 ± 1	523 ± 1	545 ± 1
